# Molecular Heterogeneity in a Patient-Derived Glioblastoma Xenoline Is Regulated by Different Cancer Stem Cell Populations

**DOI:** 10.1371/journal.pone.0125838

**Published:** 2015-05-08

**Authors:** Jo Meagan Garner, David W. Ellison, David Finkelstein, Debolina Ganguly, Ziyun Du, Michelle Sims, Chuan He Yang, Rodrigo B. Interiano, Andrew M. Davidoff, Lawrence M. Pfeffer

**Affiliations:** 1 Department of Pathology and Laboratory Medicine, and the Center for Cancer Research, University of Tennessee Health Science Center, Memphis, TN, 38163, United States of America; 2 Department of Surgery, University of Tennessee Health Science Center, Memphis, TN, 38163, United States of America; 3 Department of Pathology, St. Jude Children’s Research Hospital, Memphis, TN, 38105, United States of America; 4 Department of Computational Biology, St. Jude Children’s Research Hospital, Memphis, TN, 38105, United States of America; 5 Department of Surgery, St. Jude Children’s Research Hospital, Memphis, TN, 38105, United States of America; Swedish Neuroscience Institute, UNITED STATES

## Abstract

Malignant glioblastoma (GBM) is a highly aggressive brain tumor with a dismal prognosis and limited therapeutic options. Genomic profiling of GBM samples has identified four molecular subtypes (Proneural, Neural, Classical and Mesenchymal), which may arise from different glioblastoma stem-like cell (GSC) populations. We previously showed that adherent cultures of GSCs grown on laminin-coated plates (Ad-GSCs) and spheroid cultures of GSCs (Sp-GSCs) had high expression of stem cell markers (CD133, Sox2 and Nestin), but low expression of differentiation markers (βIII-tubulin and glial fibrillary acid protein). In the present study, we characterized GBM tumors produced by subcutaneous and intracranial injection of Ad-GSCs and Sp-GSCs isolated from a patient-derived xenoline. Although they formed tumors with identical histological features, gene expression analysis revealed that xenografts of Sp-GSCs had a Classical molecular subtype similar to that of bulk tumor cells. In contrast xenografts of Ad-GSCs expressed a Mesenchymal gene signature. Adherent GSC-derived xenografts had high STAT3 and ANGPTL4 expression, and enrichment for stem cell markers, transcriptional networks and pro-angiogenic markers characteristic of the Mesenchymal subtype. Examination of clinical samples from GBM patients showed that STAT3 expression was directly correlated with ANGPTL4 expression, and that increased expression of these genes correlated with poor patient survival and performance. A pharmacological STAT3 inhibitor abrogated STAT3 binding to the ANGPTL4 promoter and exhibited anticancer activity *in vivo*. Therefore, Ad-GSCs and Sp-GSCs produced histologically identical tumors with different gene expression patterns, and a STAT3/ANGPTL4 pathway is identified in glioblastoma that may serve as a target for therapeutic intervention.

## Introduction

Brain tumors represent an important cause of cancer-related morbidity and mortality in the United States, with malignant gliomas being among the most aggressive and difficult to treat [1]. Although they rarely metastasize, malignant gliomas are locally invasive, highly vascular tumors with extensive areas of necrosis and hypoxia. The prognosis for patients with glioma is poor. Most patients with glioblastoma multiforme (GBM), the most severe grade of glioma (WHO grade IV) and the most common glioma subtype in adults, die within 2 years of diagnosis, and patient survival has remained dismally low for decades [1]. Surgical resection of GBM remains the primary treatment modality. Present adjuvant therapies, including chemotherapy and radiation therapy, only provide slight improvement in the disease course and outcome [2]. Patients with recurrent GBM have an even bleaker prognosis [3].

Glioblastoma tumors are a heterogeneous mixture of cellular and molecular subtypes, which may underlie the inability of conventional and targeted therapies to significantly impact patient outcomes. Genomic profiling has identified four molecular subtypes of glioblastoma: Proneural, Neural, Classical and Mesenchymal [4]. The Proneural subtype is associated with PDGFRA abnormalities, IDH1 and TP53 mutations, and is usually found in younger patients. Most gliomas are classified as Proneural due to their oligodrendrocytic signature [5–7]. The gene expression of the Neural class of glioma most closely resembles normal brain tissue, and has a strong enrichment for genes differentially expressed by neurons. The Classical glioma subtype has an astrocytic signature; EGFR amplification is commonly observed in this tumor type as well as high expression of Nestin (a neural precursor and stem cell marker), and Notch and Sonic hedgehog signaling pathways. Gliomas classified as Mesenchymal exhibit higher expression of mesenchymal markers, MET and CHI3L1, and genes in the NF-κB pathway, such as TRADD, RELB and TNFSF1A, as well as deletion of NF1 [8, 9]. Tumors with a Mesenchymal gene signature tend to be more aggressive, highly resistant to therapy, lead to a higher rate of relapse and have worse overall outcomes than tumors of the Classical, Proneural and Neural subtype [8]. Therefore, a more detailed molecular understanding of the Mesenchymal subtype in GBM is crucial to improve therapeutic design and patient outcome.

The tumorigenic process in glioblastoma is apparently initiated and sustained by a rare subpopulation of GBM stem-like cells (GSCs) [10–12]. As is the case with normal stem cells, GSCs can self-renew and undergo differentiation, but they have high tumor-initiating capacity and therapeutic resistance [13]. These stem-like cells are usually isolated based on their ability to grow as multicellular, nonadherent spheres from single cell suspensions [14, 15], which we denote as spheroid GBM stem-like cells (Sp-GSCs). However, expansion of Sp-GSCs is technically challenging, and as spheres enlarge differentiated progeny appear and dying cells accumulate within the sphere’s core. As an alternative approach, adherent GSCs isolated from GBM (Ad-GSCs) and grown in chemically defined medium on laminin-coated tissue culture flasks display stem cell properties and initiate high-grade gliomas following xenotransplantation [16].

In the present report, Ad-GSCs and Sp-GSCs were isolated from a GBM patient-derived xenoline (PDX) that has the Classical gene signature. When injected subcutaneously into the flanks or orthotopically into the brains of immunocompromised mice, Ad-GSCs and Sp-GSCs were found to have markedly enhanced tumor-initiating activity (TIA) as compared to bulk tumor cells, and form tumors that display identical histological features. Of note, while both GSC populations *in vitro* have a mesenchymal gene signature, Sp-GSCs produce tumors with a Classical gene signature; thus they recapitulate the molecular properties of the original GBM PDX. In contrast, Ad-GSCs produce tumors with a mesenchymal gene signature. Besides upregulated expression of many genes typical of the mesenchymal subclass, tumors produced from Ad-GSCs showed upregulated expression of STAT3 and angiopoietin like-4 (ANGPTL4). STAT3 is an important transcription factor that plays a significant role in oncogenesis. ANGPTL4 has been reported to act not only as a tumor suppressor [17], but also as an enhancer of tumor metastasis and angiogenesis [18]. Most interestingly, a pharmacological STAT3 inhibitor blocked STAT3 binding to the ANGPTL4 promoter, and *in vivo* antitumor activity in Ad-GSC xenografts.

## Materials and Methods

### Cell culture

The human GBM6 patient-derived xenograft (PDX) of adult glioblastoma tissue was provided by Dr. C. David James, (Department of Neurological Surgery, University of California, San Francisco) [19], and continuously maintained as subcutaneous xenografts in five-week-old male NOD.Cg *Prkdc* ^*scid*^ *Il2rg* ^*tm1Wjl*^/SzJ (NSG) mice (Jackson Laboratory, Bar Harbor, ME). Cell cultures of the GBM6 PDX were derived from minced, freshly harvested tumor tissue. Short-term GBM6 cultures of differentiated bulk tumor cells were grown as adherent monolayers for 2 to 5 passages in DMEM (Cellgro, Herndon, VA) supplemented with 10% heat-inactivated fetal bovine serum (Hyclone Labs, Thermo Scientific, Rockford, IL), 100 units/mL penicillin and 100 μg/mL streptomycin. Ad-GSCs and Sp-GSCs were maintained in NeuroBasal-A medium (Invitrogen, Carlsbad, CA) containing 2% B27 supplement, 2 mM L-glutamine, 100 units/mL penicillin, 100 μg/mL streptomycin, EGF (20 ng/ml), and basic FGF (40 ng/ml). For isolation of Ad-GSCs, culture flasks were coated with 100 μg/mL poly D-lysine (Sigma-Aldrich, St. Louis, MO) for 1 hr followed by coating with 10 μg/mL laminin (Gibco, Life Technologies Inc., Grand Island NY) for 2 hr prior to use. Ad-GSCs were plated into 75 cm^2^ flasks, grown to confluence, dissociated with HyQTase (Thermo Scientific, Scientific, Rockford, IL), and split at a 1:3 ratio. For isolation of Sp-GSCs, glioma cells were dissociated with HyQtase and plated into ultra-low adhesion flasks.

### Subcutaneous xenografts

Animal experiments were performed in accordance with a study protocol approved by the Institutional Animal Care and Use Committee of the University of Tennessee Health Science Center. Glioblastoma xenografts were established in five-week-old male NOD.Cg-*Prkdc* ^*scid*^ *Il2rg* ^*tm1Wjl*^/SzJ (NSG) mice (Jackson Laboratory, Bar Harbor, ME) by direct flank injection of cells (1×10^6^) transduced with luciferase lentivirus constructs [20]. Tumors were measured twice weekly with a handheld caliper. For bioluminescence imaging, mice were injected intraperitoneally with d-luciferin (the luciferase substrate), imaged on the IVIS *in vivo* imaging system (Caliper Life Sciences, Hopkinton, MA), and photonic emissions assessed using Living image software. To determine the effect of STAT3 inhibition, once detectable tumors were determined by caliper measurement (usually within 2-weeks of cell injection), WP1066 (40 mg/kg) in DMSO/Polyethylene glycol was delivered every other day by intratumoral injection. This dose of WP1066 has been used previously in preclinical *in vivo* studies [21–23].

### Orthotopic injections

Animal studies were performed under established guidelines and supervision of the St. Jude Children’s Research Hospital’s Institutional Animal Care and Use Committee, as required by the United States Animal Welfare Act and the National Institutes of Health’s policy to ensure proper care and use of laboratory animals for research. Anesthetized (ketamine/xylazine) CB17 SCID mice were placed on stereotactic equipment where the scalp was prepped using alcohol and iodine swabs and artificial tear gel applied to the eyes. Following scalp excision, a rectangular cranial window was carved out and the dura was completely removed from the surface of the brain, and 1x10^6^ cells suspended in 10 uL of medium were injected approximately 2.5 mm deep in the right motor cortex. The excision site was closed with skin glue, and all animals were monitored closely 24 hrs post-operatively. Tumor tissue was harvested by gross inspection of the site of injection, which was easily visualized utilizing a cranial window. Once this area was identified, careful dissection allowed subtotal removal of tumor tissue alone; however, no further testing was performed to assure no mouse cells were included in the specimen.

### Gene expression analysis

Total RNA was isolated by treating tissue homogenates with Trizol followed by isolation with the RNeasy Mini kit (Qiagen Inc., Valencia, CA). Samples were submitted for complete mRNA expression profiling to the UTHSC Center of Genomics and Bioinformatics (Memphis, TN) for labeling and hybridization to Human-HT12 BeadChips (Illumina Inc.). The microarray data have been deposited in NCBI's Gene Expression Omnibus and are accessible through GEO Series accession number GSE65576 (http://www.ncbi.nlm.nih.gov/geo/query/acc.cgi?acc=GSE65576). Gene expression was also measured on the nCounter Analysis System (Nanostring Technologies, Seattle, WA) using a panel of 230 human cancer-related genes. In brief, total RNA was mixed with pairs of capture and reporter probes, hybridized on the nCounter Prep Station, and purified complexes were measured on the nCounter digital analyzer. To account for differences in hybridization and purification, data were normalized to the average counts for all control spikes in each sample and analyzed with nSolver software. Gene expression patterns were quality controlled by Principal Component Analysis (PCA). Genes were statistically tested by unequal variance t tests. The false discovery rate (FDR) was calculated to control for multiple comparisons using Partek Genomics Suite 6.6. Results were visualized using STATA/MP 11.2. In addition, 3–5 (10 μm) curls were cut from 24 glioblastoma patient biopsy specimens (UTHSC Tissue Services Core), RNA isolated using the RecoverAll Total Nucleic Acid Isolation Kit (Ambion), and gene expression was determined by quantitative RT-PCR.

### Ingenuity Pathway Analysis (IPA)

IPA (Qiagen Inc., Valencia, CA) was used to identify canonical signaling pathways and functional pathways as well as to produce networks of related genes derived from genes changed in the analyzed comparisons. Here, the rank-product-generated gene lists using a 5% FDR were uploaded into the IPA server as input data. IPA uses pathway libraries derived from the scientific literature. Statistics for functional analysis were carried out by Fischer’s exact test.

### Histopathology

Tumor tissue produced by injection of bulk tumor cells, Ad-GSCs and Sp-GSCs (four separate tumors for each condition) were fixed in 10% neutral buffered formalin for 24 hours, embedded in paraffin wax, and sectioned at 5 μm thickness. For each sample, sections were stained using a standard hematoxylin and eosin (H&E) method, or by immunohistochemistry using antibodies to neural markers: GFAP, S100, OLIG2, MAP2 and synaptophysin. Representative images of each sample/stain combination were captured at 20x original magnification on a Nikon S1 digital camera.

### Quantitative RT-PCR

Gene expression of RNA used for microarray analysis was measured by q-PCR on an iCyclerIQ (Bio-Rad Laboratories, Richmond, CA) using an iScript One-Step RT-PCR kit with SYBR Green (Bio-Rad Laboratories, Richmond, CA). Reaction parameters were as follows: cDNA synthesis at 50°C for 20 min, transcriptase inactivation at 95°C for 5 min, PCR cycling at 95°C for 10 sec, and 60°C for 30 sec for 40 cycles. The following primers were used for RT-PCR: β-actin 5’-AGAAGGAGATCACTGCCCTG-3′ (forward), 5’-CACATCTGCTGGAAGGTGGA-3′ (reverse); CHI31 5’-GTGAAGGCGTCTCAAACAGG-3’ (forward), 5’-GAAGCGGTCAAGGGCATCT-3’ (reverse); TRADD 5’-GCTGTTTGAGTTGCATCCTAGC-3’ (forward), 5’-CCGCACTTCAGATTTCGCA-3’ (reverse); NF1 5’-AGATGAAACGATGCTGGTCAAA-3’ (forward), 5-CCTGTAACCTGGTAGAAATGCGA-3’ (reverse); RelB 5’-CAGCCTCGTGGGGAAAGAC-3’ (forward), 5’-GCCCAGGTTGTTAAAACTGTGC-3’ (reverse); CASP4 5’-TTTCTGCTCTTCAACGCCACA-3’ (forward), 5’-AGCTTTGGCCCTTGGAGTTTC-3’ (reverse); FGFR3 5’-TGCGTCGTGGAGAACAAGTTT-3’ (forward), 5’-GCACGGTAACGTAGGGTGTG-3’ (reverse); PDGFA 5’-GCAAGACCAGGACGGTCATTT-3’ (forward), 5’-GGCACTTGACACTGCTCGT-3’ (reverse); EGFR 5’-CTACGGGCCAGGAAATGAGAG-3’ (forward), 5’-TGACGGCAGAAGAGAAGGGA-3’ (reverse); AKT2 5’-ACCACAGTCATCGAGAGGACC-3’ (forward), 5’-GGAGCCACACTTGTAGTCCA-3’ (reverse); Nestin 5’-GGCGCACCTCAAGATGTCC-3’ (forward), 5’- CTTGGGGTCCTGAAAGCTG-3’ (reverse).

### TCGA data analysis

To examine the relationship between STAT3 and ANGPTL4 expression in human GBM brain tissue, we queried the TCGA data portal for all low-grade glioma and GBM samples with gene expression (BI_HT_HG-U113A Array Data Set) data available as well as accompanying clinical data. The data set was filtered for samples having expression data for STAT3, ANGPTL4 and clinical data, yielding a final set of 466 individual low-grade glioma samples and 328 independent GBM patient samples. Statistical analysis was performed using Graphpad Prism.

### Apoptosis assay

The induction of apoptosis was monitored by flow cytometry (Accuri Model 6C) using the Annexin V-FITC apoptosis detection kit (BD Pharmingen, San Diego, CA), according to the manufacturer's instructions.

### Chromatin immunoprecipitation

Chromatin Immunoprecipitation (ChIP) was carried out using the ChIP-I Express Enzymatic kit (Active Motif, Carlsbad, CA) according to the manufacturer's instructions. In brief, chromatin from cells was cross-linked with 1% formaldehyde (10 min at 22°C), sheared to an average size of ~200 bp, and then immunoprecipitated with anti-STAT3 (Santa Cruz Biotechnology). ChIP-PCR primers were designed to amplify a proximal promoter region containing a putative STAT3 (-1369 to -1348) binding site in the ANPTL4 promoter. The primers used were 5’- CATTAAAGACCCTGGCGGTA -3’ (forward), 5’- GGATCACAGTCGTGTGAGGA -3’ (reverse).

### Statistical analysis

At least three independent experiments were performed in duplicate, and data are presented as means ± sd. ANOVA and post-hoc least significant difference analysis or Student *t* tests were performed. *p* values < 0.05 (*) were considered statistically significant.

## Results

### Differences in the molecular signatures of GSCs grown *in vitro* and as subcutaneous xenografts

**Fig 1:**
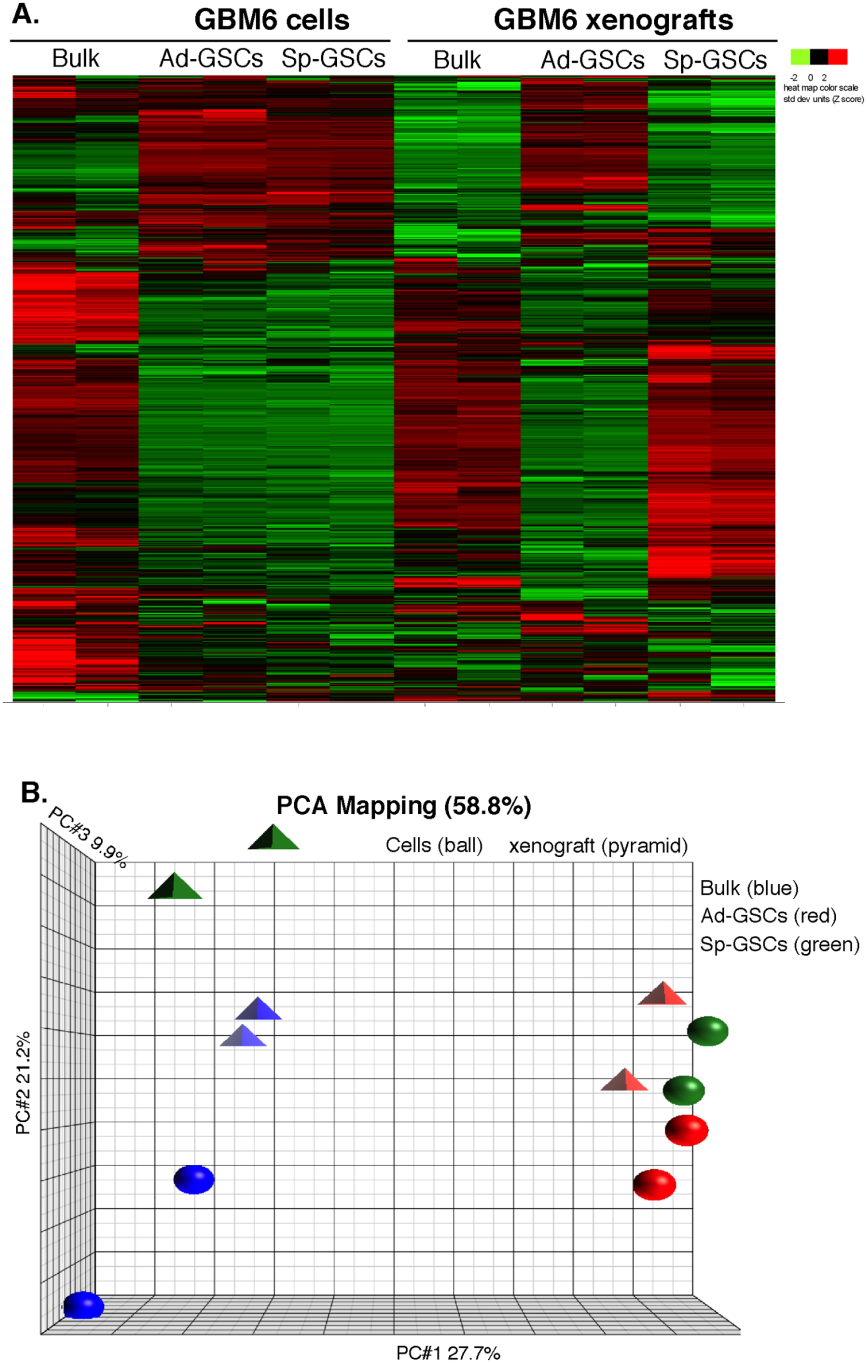
Microarray analysis of gene expression in GBM6 cells and tumor xenografts. RNA was prepared from GBM6 bulk tumor cells, Ad-GSC, and Sp-GSC cultures as well as from subcutaneous tumor xenografts of these injected cells. Biological duplicates were run for each sample and data were collected and analyzed. A. Gene expression was measured by Illumina array and genes reported in the TCGA database were analyzed. B. The PCA plot represents the comparison of gene signatures from each condition.

Glioblastoma is characterized by extensive heterogeneity at the cellular and molecular levels [24], which may reflect the presence of different cancer stem cell populations. In a previous study, we isolated Ad-GSCs and Sp-GSCs from the GBM6 PDX, and showed that both populations had high expression of stem cell markers, such as CD133, Sox2 and Nestin, but low expression of differentiation markers such as βIII-tubulin and glial fibrillary acid protein as compared to bulk tumor cells [25]. Both populations maintain high CD133 expression (>85%) by flow cytometric analysis even when they were maintained in serum-containing media for a week, which provides strong evidence that these GSCs represent a “true” stem cell population and that “stemness” is not an artifact of the media (serum versus growth factors). Both populations had high constitutive STAT3 and NF-κB activation, which resulted in upregulation of the Notch pathway. To more fully characterize the molecular signatures of these GSC populations, RNA was prepared from biological replicates of GBM6 cells grown as either short term cultures of differentiated bulk tumor cells, Ad-GSCs, or Sp-GSCs, and whole genome expression profiling was performed on HT-12 expression Bead-Chips by the UTHSC Center of Genomics and Bioinformatics. Hierarchical clustering was used to assess the differential expression of ~840 GBM subtype predictor genes [4]. As shown in Fig 1A, a heatmap of the variation in expression of these predictor genes showed that cells grown *in vitro* under both GSC conditions exhibited very similar expression profiles, which was markedly different from the expression profile of the highly differentiated bulk tumor cells grown in serum-containing medium. Consistent with our previous findings [25], high Nestin, Sox2 and CD133 expression was found in both GSC populations, while βIII-tubulin and glial fibrillary acid protein was expressed at relatively low levels. Thus, although the two GSC populations were grown under different culture conditions (adherent versus suspension culture), their gene expression profiles were very similar. We then compared the mathematical variance among the various data samples by PCA, which is an unsupervised analytical method similar to factor analysis that is sensitive to all causes of variability within the data. Visualization of the first three components allows for quality control and the relative assessment of the variability of replicates. This PCA visualization also allows the assessment of the categorical factors of interest by demonstrating whether the data naturally aggregates by these factors or by an unknown or systematic factor like batch. When gene expression of the GSC populations and bulk tumor cells grown *in vitro* were subjected to PCA analysis, the gene expression profiles in the two different GSC cultures were also found to be relatively similar, while that of bulk tumor cells grown *in vitro* was markedly different (Fig 1B).

We then performed whole genome expression analysis on tumors produced from the different GBM cell populations. In brief, bulk tumor cells, Ad-GSCs and Sp-GSCs (1 x 10^6^ cells) were injected into the flanks of immunocompromised NSG mice, and once reaching a volume of ~200 mm^3^, tumors were excised, RNA was prepared and subjected to microarray analysis. In contrast to the *in vitro* findings, tumors produced from Ad-GSCs and Sp-GSCs had markedly different expression profiles as evidenced by the heatmaps of the gene expression profiles (Fig 1A) as well as PCA of the average gene expression (Fig 1B). The tumor xenografts of the differentiated bulk tumor cells and Sp-GSCs were extremely similar, which is consistent with the finding that Sp-GSCs repopulate the GBM tumors with gene expression profiles nearly identical to that of bulk tumor cells [26]. Therefore, while the gene expression of Sp-GSCs and Ad-GSCs grown *in vitro* are highly similar, the *in vivo* molecular signature of the tumor tissue is quite different. These results suggest that distinct stem cell populations exist in GBM and promote tumor heterogeneity.

### Ad-GSC and Sp-GSC xenografts express distinct molecular profiles

**Fig 2:**
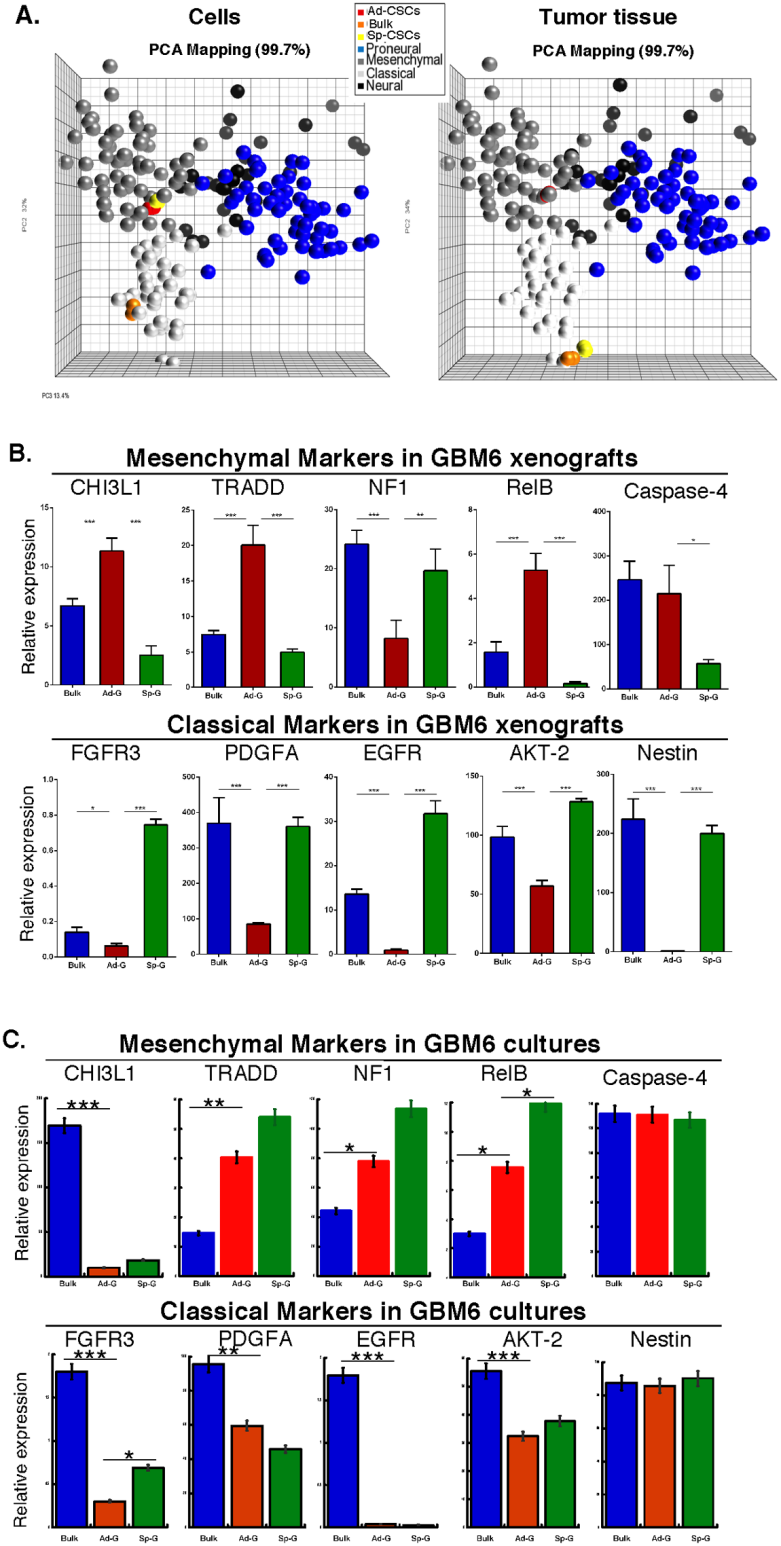
Molecular classification of gene expression in GBM6 cells and tumor xenografts. A. Array analysis was performed and compared to glioblastoma molecular subclasses; PCA map depicts the classical, mesenchymal, neural, and proneural gene signatures and the gene signature of GBM6 cells and tumor tissue derived from bulk tumor cells, Ad-GSCs and Sp-GSCs. B. and C. RNA was isolated from three individual tumors derived from GBM6 bulk tumor cells, Ad-GSCs and Sp-GSCs (B), and from these cells grown *in vitro* (C.) to determine gene expression of molecular markers of the Mesenchymal (CHI3L1, TRADD, NF1, RelB and CASP4) and Classical (FGFR3, PDGFA, EGFR, AKT2 and Nestin) subclass of glioblastoma by qPCR and normalized to actin expression (n = 3). Error bars, S.D. * p < 0.05, ** p < 0.01, *** p < 0.001.

To determine if the molecular profiles of Ad-GSCs and Sp-GSCs corresponded to any of the four molecular subtypes of glioblastoma we compared the expression of the ~840 GBM predictor genes in our cell and tissue samples to that of 202 glioblastoma tumor tissue in the TCGA database, which represent Classical (white spheres), Neural (black spheres), Proneural (blue spheres) and Mesenchymal (grey spheres) subclasses. Fig 2A shows, as expected, that the GBM tumor samples form four relatively distinct groups as previously shown [4], but there is some overlap in the expression patterns among these four subclasses. The gene expression profiles of Ad-GSCs (red spheres) and Sp-GSCs (yellow spheres) grown *in vitro* resemble that of the Mesenchymal GBM subclass. In contrast the expression profile of bulk GBM6 tumor cells grown *in vitro* (orange spheres) is associated with the Classical GBM subtype, which is consistent with what was previously found (Y. Gillespie, personal communication). We next examined the pattern of gene expression in flank tumors of these cells. Most interestingly, tumors that arose from Ad-GSCs expressed a Mesenchymal gene signature, which resembles that of Ad-GSCs grown *in vitro*. In contrast, tumors that arose in mice injected with Sp-GSCs had a Classical subtype signature, which closely resembles the gene signature of tumors derived from bulk tumor cells. Thus, Sp-GSCs and Ad-GSCs have similar molecular properties (Mesenchymal subclass) *in vitro*, which are distinct from that of bulk tumor cells grown (Classical subclass) *in vitro*. When injected into mice, these GSCs form molecularly distinct tumors. Ad-GSCs maintain a Mesenchymal gene signature *in vitro* and *in vivo*, while Sp-GSCs repopulate the tumor with a Classical gene signature, similar to that of bulk tumor cells.

To further characterize gene expression in the Ad-GSCs and Sp-GSCs tumor xenografts, we examined the expression of genes previously defined to be characteristic of the Classical (FGFR3, PDFA, EGFR, AKT-2 and Nestin) and Mesenchymal (CHI3L1, TRADD, NF1, RelB and CASP4) glioblastoma subtypes [4]. RNA was extracted and pooled from three individual subcutaneous xenografts derived from bulk tumor cells, Ad-GSCs or Sp-GSCs, and the expression of these marker genes was determined by qPCR. Fig 2B shows that expression of Mesenchymal markers CHI3L1, TRADD and RelB is significantly elevated in Ad-GSCs xenografts as compared to tumor xenografts of bulk tumor cells and Sp-GSCs. In contrast, the tumor suppressor NF1, which is downregulated in Mesenchymal glioblastoma, is expressed at relatively low levels in tumor tissue from Ad-GSCs as compared to tumors derived from Sp-GSCs and bulk tumor cells. The Mesenchymal marker, CHI3L1, in combination with astrocytic markers is indicative of an epithelial-to-mesenchymal transition that has been linked to aggressive, dedifferentiated tumors [27]. Genes in the TNF and NF-κB pathways, such as TRADD and RelB, are highly expressed in the Mesenchymal subtype as well, potentially as a consequence of increased necrosis and associated inflammatory infiltrates [28]. In addition, the Classical marker genes FGFR3, PDGFA, EGFR and Nestin are all expressed at relatively high levels in tumor xenografts of bulk tumor cells and Sp-GSCs, as compared to xenografts of Ad-GSCs, which is consistent with the PCA analysis classification of the tumors as the Classical subtype. In contrast, EGFR and Nestin are expressed at extremely low levels in Ad-GSC tumor xenografts, which is consistent with their classification as Mesenchymal tumors. Significant EGFR amplification is observed in 97% of Classical glioblastomas in the TCGA database but infrequently in other subtypes. The GBM6 xenograft is derived from a patient with overexpression of the VIII mutant of EGFR, and our finding of high EGFR expression in bulk tumor cells and in tumors derived from them is consistent with EGFR overexpression. However, it is extremely interesting that the tumors from Ad-GSCs express relatively low EGFR levels providing additional evidence that Ad-GSCs are a distinct GSC population. Based on these findings on differential marker gene expression in GSC xenografts, we then examined the expression of the Mesenchymal and Classical marker genes in bulk tumor cells, Ad-GSCs and Sp-GSCs grown *in vitro*. As shown in Fig 2C, the expression of the Classical marker genes FGF3, PDGFA, EGFR and AKT2 is markedly downregulated in both GSC populations relative to bulk tumor cells grown *in vitro*. In addition, there is elevated expression of the Mesenchymal marker genes TRADD, NF1 and RelB in both Ad-GSC and Sp-GSC populations grown *in vitro*. Overall our studies indicate that Ad-GSCs are a molecularly distinct GSC subpopulation from that of Sp-GSCs. The Ad-GSCs exhibit a Mesenchymal gene signature *in vitro*, and promote the formation of Mesenchymal tumors *in vivo*.

### Histopathology of Sp-GSC and Ad-GSC xenografts

**Fig 3:**
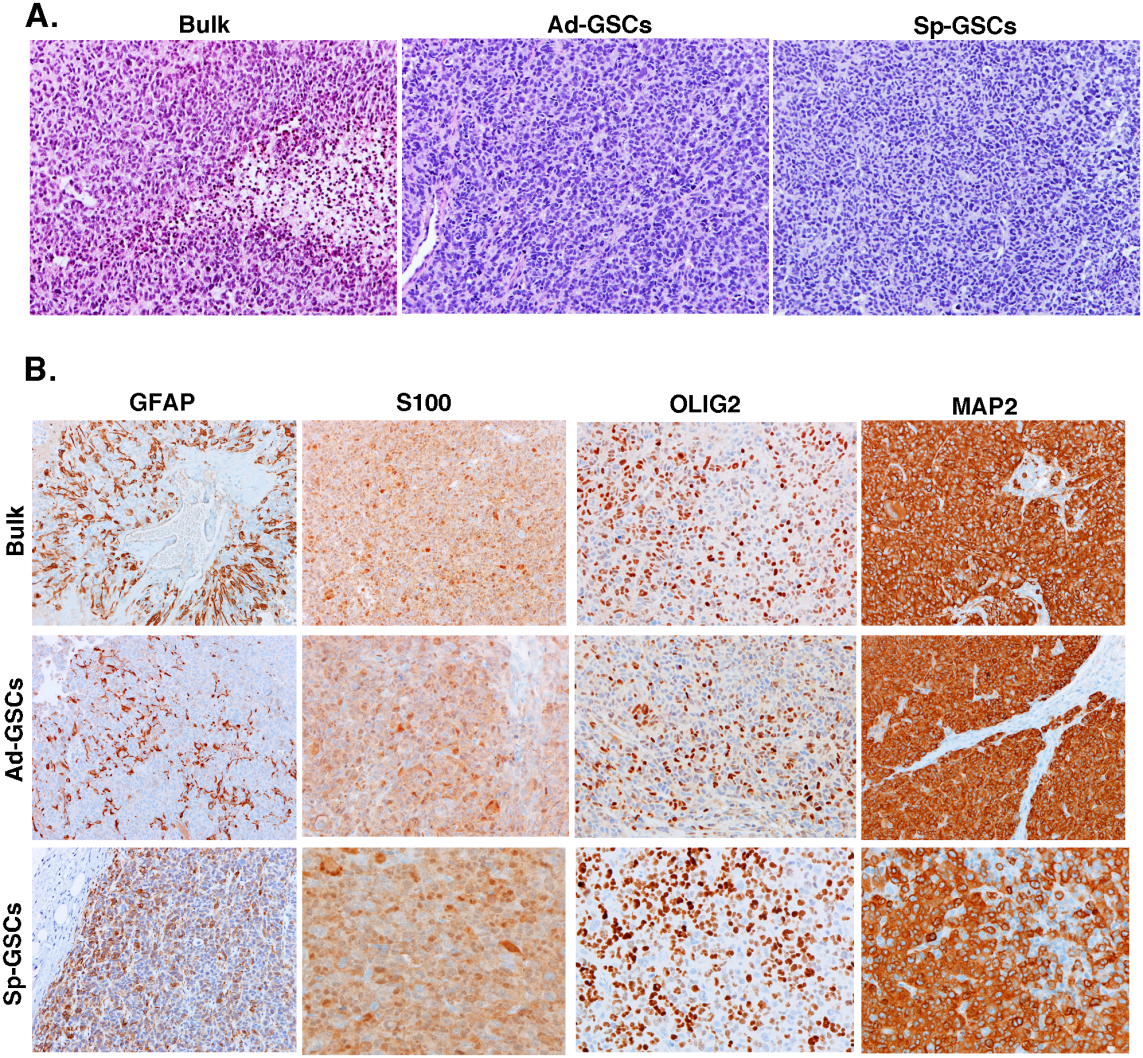
Pathologic analysis of subcutaneous GBM6 tumor xenografts. GBM6 bulk tumor cells, Ad-GSCs, and Sp-GSCs were injected subcutaneously and allowed to grow to a diameter of ~400mm^3^. Tumors were harvested and paraffin embedded for histology. A. H&E staining and B. Immunoreactivity for GFAP, S100, OLIG2 and MAP2. (All photomicrographs taken at 200x).

An important characteristic of GBM is marked morphologic heterogeneity within the tumor itself, as well as among different GBM tumors. The molecular heterogeneity observed in our glioblastoma xenografts of bulk tumor cells, Ad-GSCs and Sp-GSCs led us to examine the histopathology of these tumors. Four individual subcutaneous tumors derived from each cell culture condition were formalin-fixed, paraffin-embedded, and sectioned. Each sample was H&E stained, and immunohistochemistry was performed to measure immunoreactivity with antibodies for the following neural markers: GFAP, S100, OLIG2, MAP2 and synaptophysin. As shown in Fig 3A, the Classical GBM tumors of bulk tumor cells and Sp-GSCs xenografts, and the Mesenchymal tumors of Ad-GSC xenografts were all determined to be high-grade gliomas and were morphologically indistinguishable. Tumor cells with a high nuclear:cytoplasmic ratio and little nuclear pleomorphism showed the morphology of relatively undifferentiated high-grade gliomas. Immunohistochemistry of the tumor tissue demonstrated a uniform phenotype among the different tumor xenografts (Fig 3B). There was strong immunoreactivity for GFAP and OLIG2 in many tumor cells, while all tumor cells expressed S-100 and MAP-2. There was no immunoreactivity for synaptophysin. Across the range of neural tumors, GFAP, OLIG-2 and MAP-2 are generally expressed by gliomas, while the neuronal marker synaptophysin is not [29–32]. These findings reveal the similarity at the microscopic level between the molecularly distinct xenografts of the bulk tumor cells, Sp-GSCs and Ad-GSCs.

### Adherent GSCs promote the formation of intracranial tumors with a Mesenchymal signature

**Fig 4:**
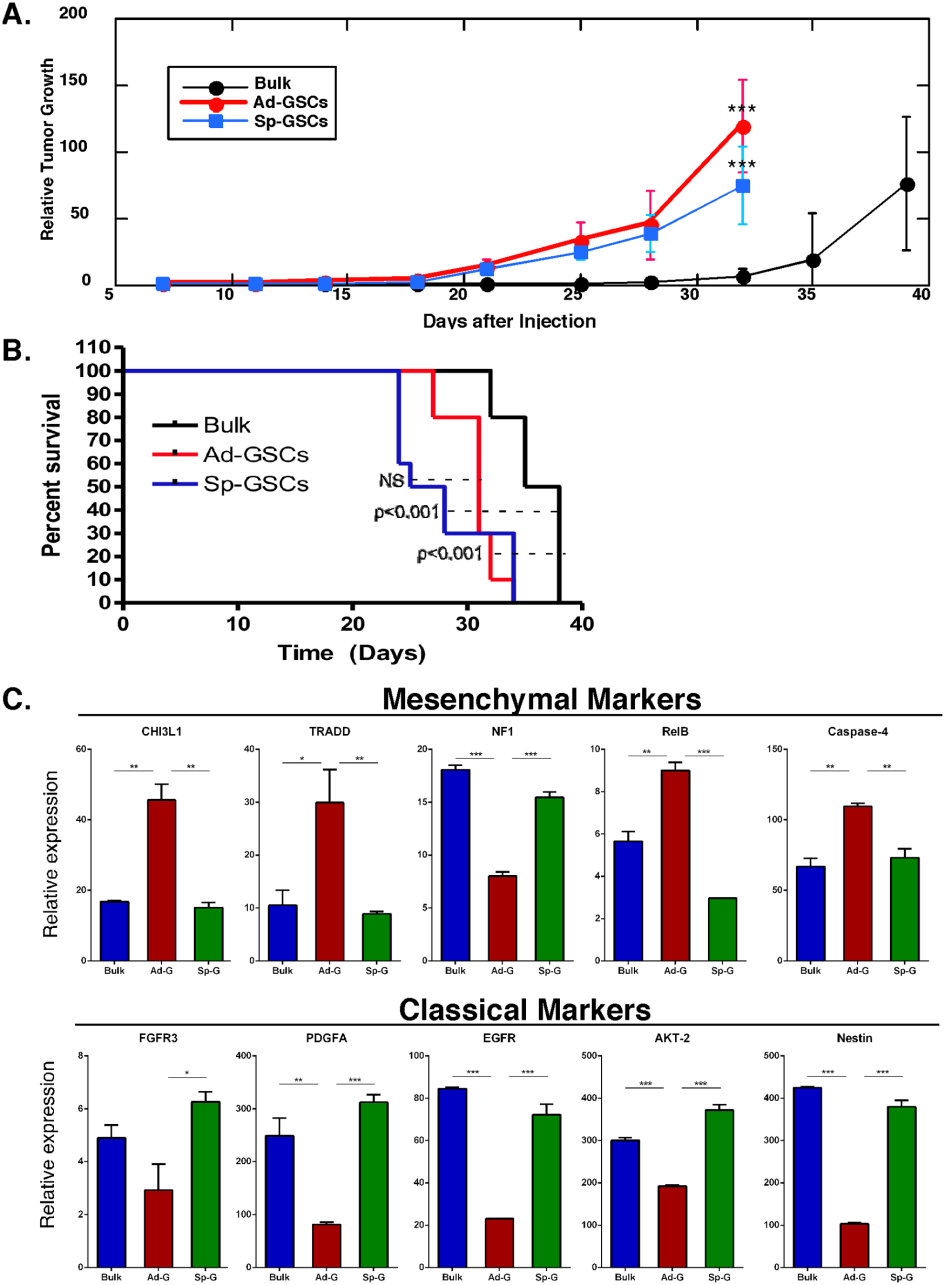
Characterization of GBM6 tumor xenografts. A. CB17 SCID mice were injected with 1x10^6^ luciferase-tagged GBM6 cells grown as bulk tumor cells, Ad-GSCs, or Sp-GSCs, and tumor burden was measured by bioluminescence twice a week (n = 10 per group), and (B) Kaplan-Meier analysis of survival data (n = 8) was performed. C. RNA isolated from intracranial tumors was subjected to qPCR as indicated and normalized to actin expression (n = 3). Error bars, S.D. * p < 0.05, ** p < 0.01, *** p < 0.001.

In order to investigate the role of the tumor microenvironment on gene expression in GBM, we also performed intracranial injection of GSCs. The human GBM orthotopic mouse model results in invasive growth in mice and allows quantitation of intracranial tumor growth [33]. We first compared the tumor initiating activity of luciferase-expressing GBM6 cells grown as short-term bulk tumor cells, Ad-GSCs or Sp-GSCs by bioluminescence imaging. Fig 4A shows that both Ad-GSCs and Sp-GSCs formed intracranial tumors more rapidly than bulk tumor cells (within 25 days as compared to 35 days, respectively). Moreover, animal survival was significantly (p <0.001) shorter with the more aggressive tumors (Fig 4B). This finding is consistent with our previous studies that revealed Ad-GSCs were more potent in inducing subcutaneous tumors when compared to bulk GBM tumor cells [25].

**Fig 5:**
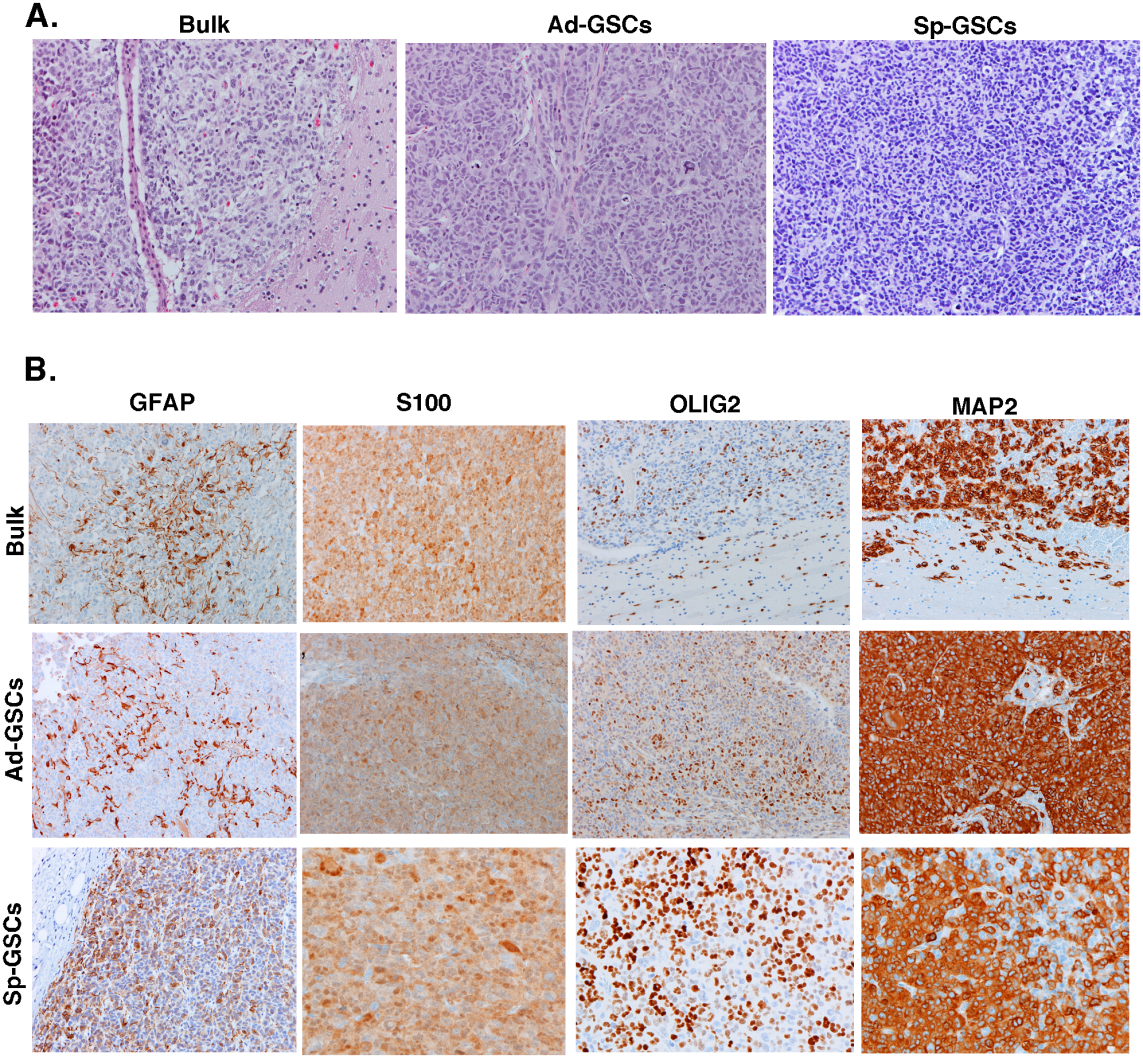
Pathologic analysis of orthotopic GBM6 tumor xenografts. GBM6 bulk tumor cells, Ad-GSCs and Sp-GSCs were orthotopically injected as described in Fig 4, and tumor-bearing brains were paraffin embedded for histology. A. H&E staining and B. Immunoreactivity for GFAP, S100, OLIG2 and MAP2. (All photomicrographs taken at 200x).

We next determined the histology of the different intracranial xenografts. As shown in Fig 5A, the intracranial glioblastoma tumors derived from bulk tumor cells, Ad-GSCs and Sp-GSCs were all determined to be high-grade glioma and exhibited no distinction in H&E staining. All of the tissue displayed characteristics of glioblastoma, such as hypercellularity, atypical nuclei, pseudopalisading necrosis and microvascular proliferation [34]. The traditional glioblastoma prognostic markers GFAP, S100, OLIG2, MAP2 and SYN were also analyzed and found to be indistinguishable among the intracranial glioblastoma xenografts (Fig 5B). Thus, the intracranial and subcutaneous tumor xenografts were indistinguishable histologically.

To further characterize the intracranial glioblastoma xenografts, we also examined the expression of genes typical of the Classical and Mesenchymal GBM subtypes. RNA was extracted from three individual intracranial tumors derived from bulk tumor cells, Ad-GSCs or Sp-GSCs. As shown in Fig 4C, expression of the Mesenchymal markers CHI3L1, TRADD and RelB was significantly elevated in intracranial tumors derived from Ad-GSCs compared to the other xenografts, while NF1 was decreased. These results are similar to our findings with subcutaneous tumors (Fig 2B). In addition, we found that the Classical markers FGFR3, PDGFA, EGFR, AKT-2 and Nestin are highly expressed in intracranial tumor tissue derived from bulk tumor cells and Sp-GSCs, while there is lower expression in Ad-GSC intracranial tumors. These orthotopic studies support our finding that Ad-GSCs represent a different tumor initiating subpopulation than Sp-GSCs. The adherent glioblastoma GSCs exhibit a Mesenchymal gene signature and produce tumors of the Mesenchymal glioblastoma subtype *in vivo*.

### Upregulated expression of STAT3 and ANGPTL4 in Ad-GSC xenografts

**Fig 6:**
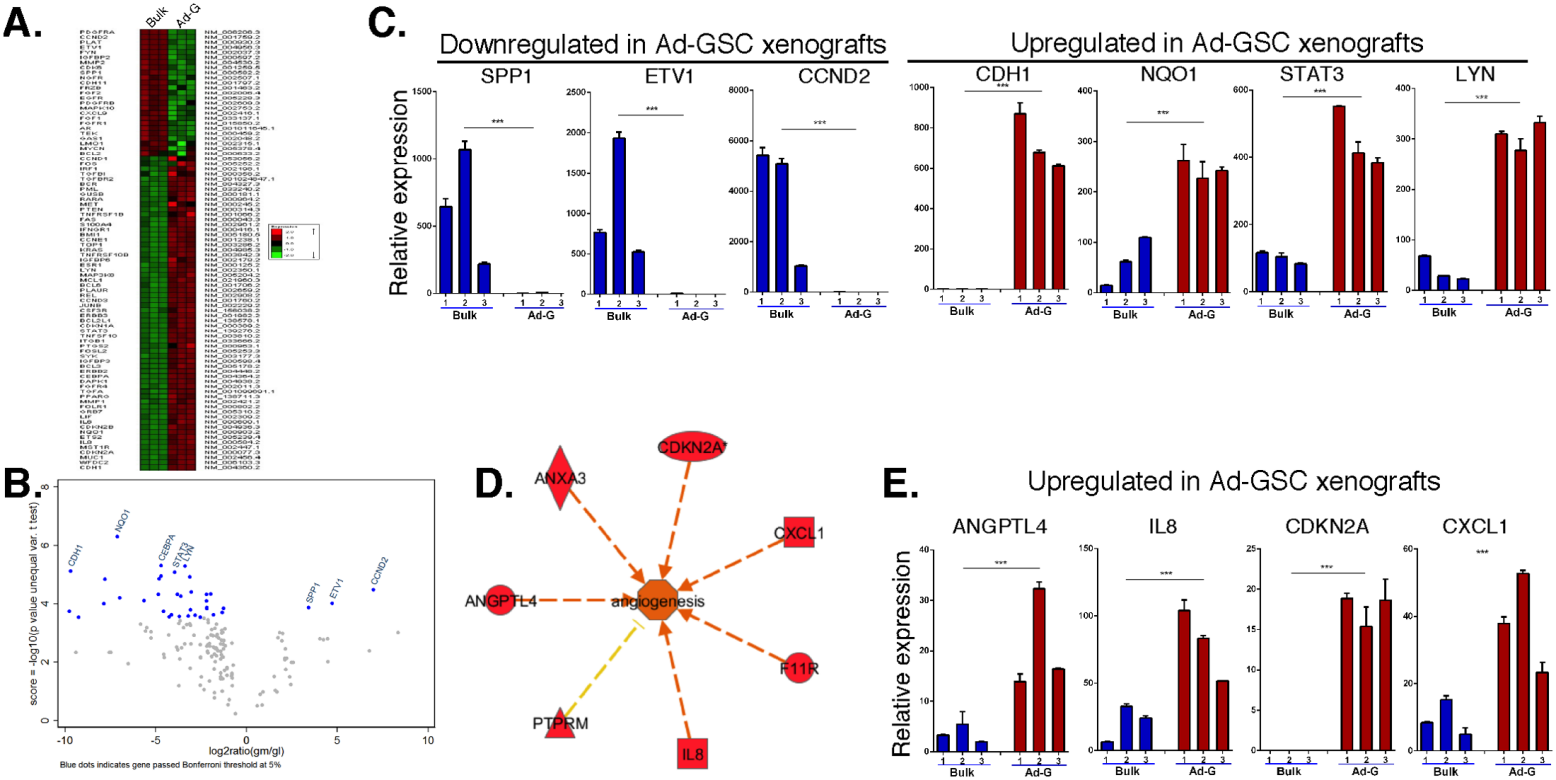
Enrichment of pro-survival and pro-angiogenic genes in Ad-GSC tumor xenografts. A. RNA was prepared from three separate tumors generated from GBM6 bulk tumor cells and Ad-GSCs, and Nanostring analysis was performed. B. Volcano plot depicting the genes that passed the Bonferroni test. C. The RNA was used to validate the gene expression of SPP1, ETV1, CCND2, CDH1, NQO1, and LYN by qPCR (normalized to actin expression). D. Ingenuity pathway analysis showing that genes upregulated in Ad-GSC tumor xenografts are involved in angiogenesis. E. qPCR validation of pro-angiogenic genes (ANGPTL4, IL8, CDKN2A and CXCL1) upregulated in Ad-GSC tumor xenografts normalized to actin expression (n = 3). Error bars, S.D. * p < 0.05, ** p < 0.01, *** p < 0.001.

To identify genes in oncogenic pathways differentially expressed in Ad-GSC xenografts, we performed expression profiling using the human cancer-related panel on the nCounter Analysis System (Nanostring Technologies, Seattle, WA). RNA was prepared from three individual subcutaneous tumors from NSG mice injected with bulk tumor cells or Ad-GSCs. Fig 6A reveals that, while individual biological replicates of tumor tissue showed little variation in gene expression, there were clear differences in the gene expression pattern in xenograft tumors of bulk tumor cells as compared to Ad-GSCs. Genes downregulated (SPP1, ETV1, CCND2) or upregulated (CDH1, NQO1, STAT3 and LYN) in the Ad-GSC-derived tumors that passed the Bonferroni threshold are shown in Fig 6B by Volcano Plots. The differential expression of these genes was validated by qPCR in three individual subcutaneous tumors derived from bulk tumor cells and Ad-GSCs (Fig 6C). The enhanced expression of STAT3 in Ad-GSC derived tumors is of particular importance, since STAT3 has been reported to be an initiator and master regulator of mesenchymal transformation in glioblastoma [35]. Thus, it is consistent that STAT3 is upregulated in Ad-GSC xenograft tumors that have a Mesenchymal expression pattern [35]. Elevated expression of CDH1, NQO1 and LYN has also been previously identified in glioblastoma, and is believed to contribute to the growth and invasion of this aggressive brain tumor subtype [36–38].

Ingenuity Pathway Analysis revealed that genes involved in angiogenesis are significantly enriched (P<0.05) in Ad-GSC tumor xenografts (Fig 6D). Fig 6E shows the qPCR validation of several pro-angiogenic genes, such as ANGPTL4, IL8, CDKN2A and CXCL1, in Ad-GSC tumor xenografts. Upregulation of ANGPTL4 expression was previously identified in an angiogenic gene signature that correlates with the Mesenchymal subtype of glioblastoma [8]. The chemokine IL-8 is expressed and secreted at high levels in glioblastoma both *in vitro* and *in vivo*, and recent experiments suggest it is critical to glial tumor neovascularity and progression [39]. CXCL1 is a chemokine implicated as an oncogenic factor in glioma, which is associated with attenuated angiogenic activity through NF-κB regulation [40]. These pro-angiogenic genes have been shown to contribute to the vascularization of highly aggressive glioblastoma tumors and therefore are of therapeutic interest.

### STAT3 and ANGPTL4 expression is associated with glioma grade and patient survival in clinical specimens

**Fig 7:**
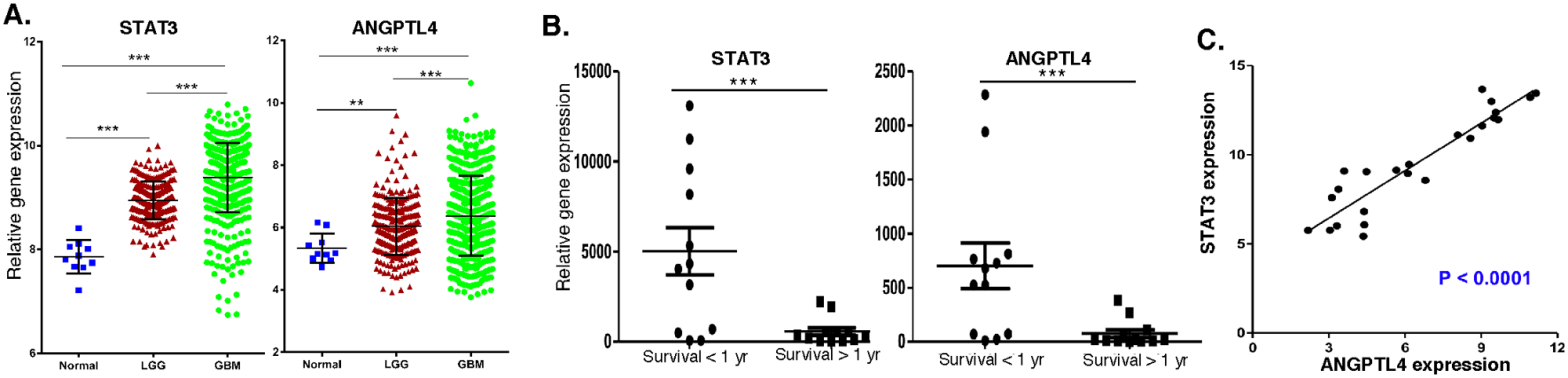
Expression of STAT3 and ANGPTL4 in human GBM samples. A. Gene expression of STAT3 and ANGPTL4 in the TCGA database for normal brain tissue (n = 10), low-grade glioma samples (n = 265) and high-grade (GBM) samples (n = 328). B. and C. RNA was extracted from 24 GBM patient biopsies, and STAT3 and ANGPTL4 expression was measured by qPCR and normalized to actin expression (n = 3), and expression was correlated to short-term and long-term survival (B) and (C) as well as to each other. Error bars, S.D. * p < 0.05, ** p < 0.01, *** p < 0.001.

We then analyzed whether the expression of STAT3 and ANGPTL4 was correlated with glioma grade in the TCGA database. STAT3 and ANGPTL4 expression was examined in 10 normal brain samples, 466 individual low-grade glioma patients and 328 individual high-grade (GBM) patients. As shown in Fig 7A, both STAT3 and ANGPTL4 expression is significantly increased in GBM samples when compared to normal tissue and low-grade gliomas.

We then determined whether the expression of STAT3 and ANGPTL4 correlates with patient survival. Brain biopsy specimens from 24 GBM patients were obtained from the UTHSC tissue core, and RNA was isolated from FFPE tissue blocks to determine expression of STAT3 and ANGPTL4. As shown in Fig 7B, although there was significant patient-to-patient variability as expected, there was a direct relationship between STAT3 and ANGPTL4 expression and patient survival. A statistically significant increase in STAT3 and ANGPTL4 gene expression was observed in GBM patients that survived less than one year compared to patients that survived longer than one-year. Interestingly, patients that exhibited highly elevated expression of STAT3 also had highly elevated ANGPTL4 expression, while other patients were found to coexpress low levels of both genes. Fig 7C shows that expression of STAT3 and ANGPTL4 are directly correlated to one another.

### STAT3 binds to the ANGPTL4 promoter and regulates ANGPTL4 expression

**Fig 8:**
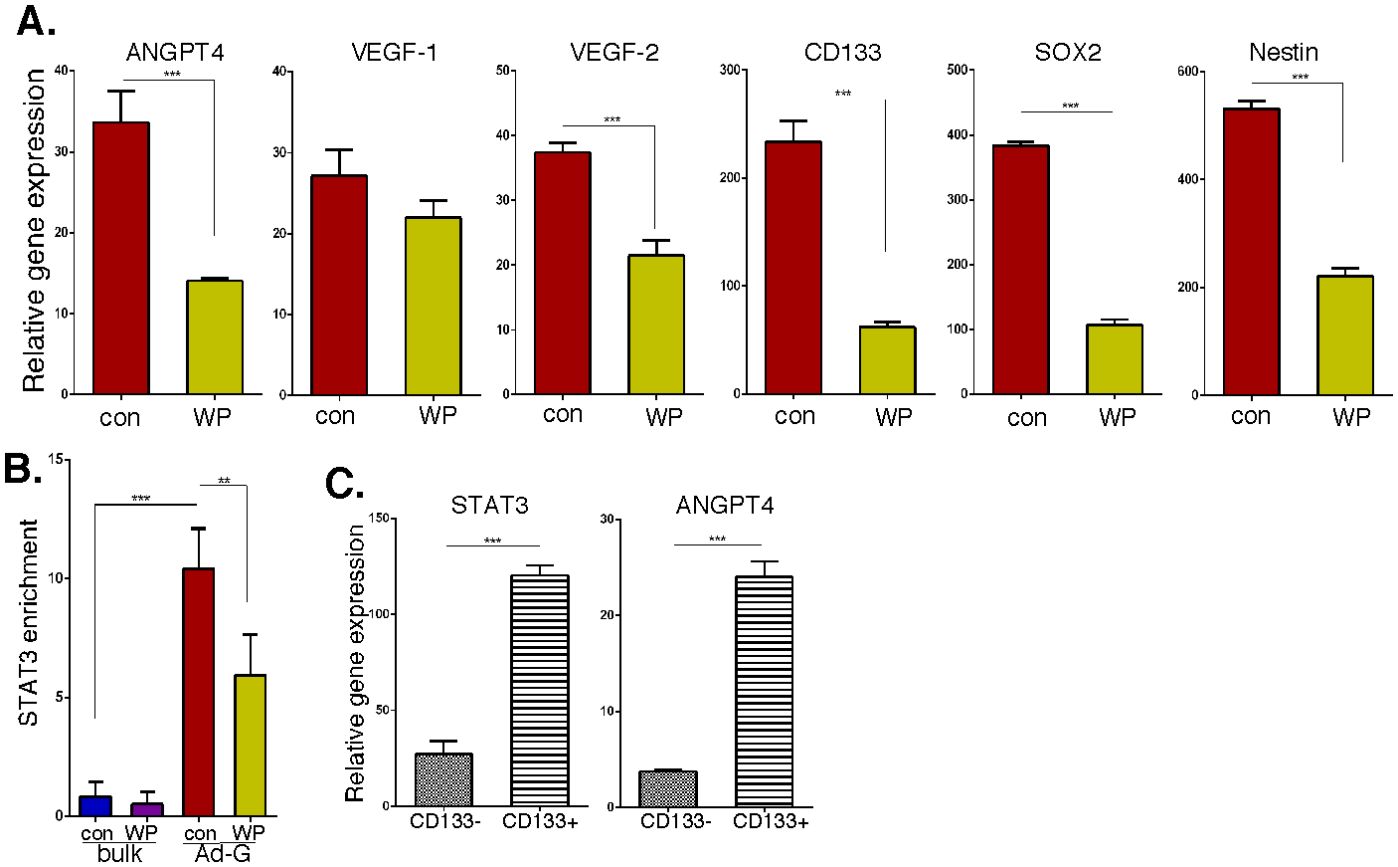
The STAT3/ANGPTL4 pathway in GSCs. A. RNA was prepared from GBM6 Ad-GSCs treated with WP1066 (50 μM) for 6 hrs, and the expression of ANGPTL4, VEGF-R1, VEGF-R2, CD133, SOX2 and Nestin was quantified by qPCR and normalized to actin expression (n = 3). B. ChIP-enriched STAT3 binding to the ANPTL4 promoter was analyzed by qPCR and normalized to input DNA, followed by subtraction of nonspecific binding determined by control IgG. C. Single cells isolated from bulk GBM6 tumor cells were incubated with anti-CD133 antibody, and data acquisition and analysis was performed on a three-laser LSR (Becton-Dickinson) flow cytometer using CellQuest software. RNA isolated from CD133+ and CD133- cells was subjected to qPCR as indicated and normalized to actin expression. Error bars, S.D. * p < 0.05, ** p < 0.01, *** p < 0.001.

To characterize the functional importance of upregulated STAT3 expression, we examined the effects of treatment with WP1066, which is a STAT3 inhibitor [25], on gene expression in Ad-GSCs. As shown in Fig 8A, treatment with WP1066 decreased the expression of angiogenic genes (ANGPTL4, VEGFR-1 and VEGFR-2), as well as expression of stem cell marker genes (CD133, SOX2 and Nestin). Since STAT3 and ANGPTL4 expression are directly correlated in GBM patient samples, and a STAT3 inhibitor suppressed ANGPTL4 expression *in vitro*, we hypothesized that STAT3 may directly regulate ANGPTL4 expression. Examination of putative transcription binding sites within the ANGPTL4 promoter revealed a proximal (-1369 to -1348) STAT3 binding site. To determine whether STAT3 bound to this site we performed ChIP analysis. As shown in 8B, significantly increased binding of STAT3 to the ANGPTL4 promoter was observed in Ad-GSCs as compared to bulk tumor cells. In addition, the STAT3 inhibitor WP1066 decreased STAT3 binding to ANGPTL4 in Ad-GSCs. Taken together, these results show that STAT3 regulates ANGPTL4 expression in Ad-GSCs by directly binding to its promoter, and this interaction can be blocked with targeted STAT3 inhibitors. As an independent approach to define whether there is upregulation of the STAT3/ANGPTL4 pathway in GSCs, we flow-sorted single cells isolated from PDX tissue for the expression of CD133 and determined STAT3 and ANGPTL4 expression by qPCR. As shown in Fig 8C, expression of STAT3 and ANGPTL4 was markedly increased in CD133+ cells as compared to CD133- cells.

### The *in vivo* antiglioma effects of STAT3 inhibition

**Fig 9:**
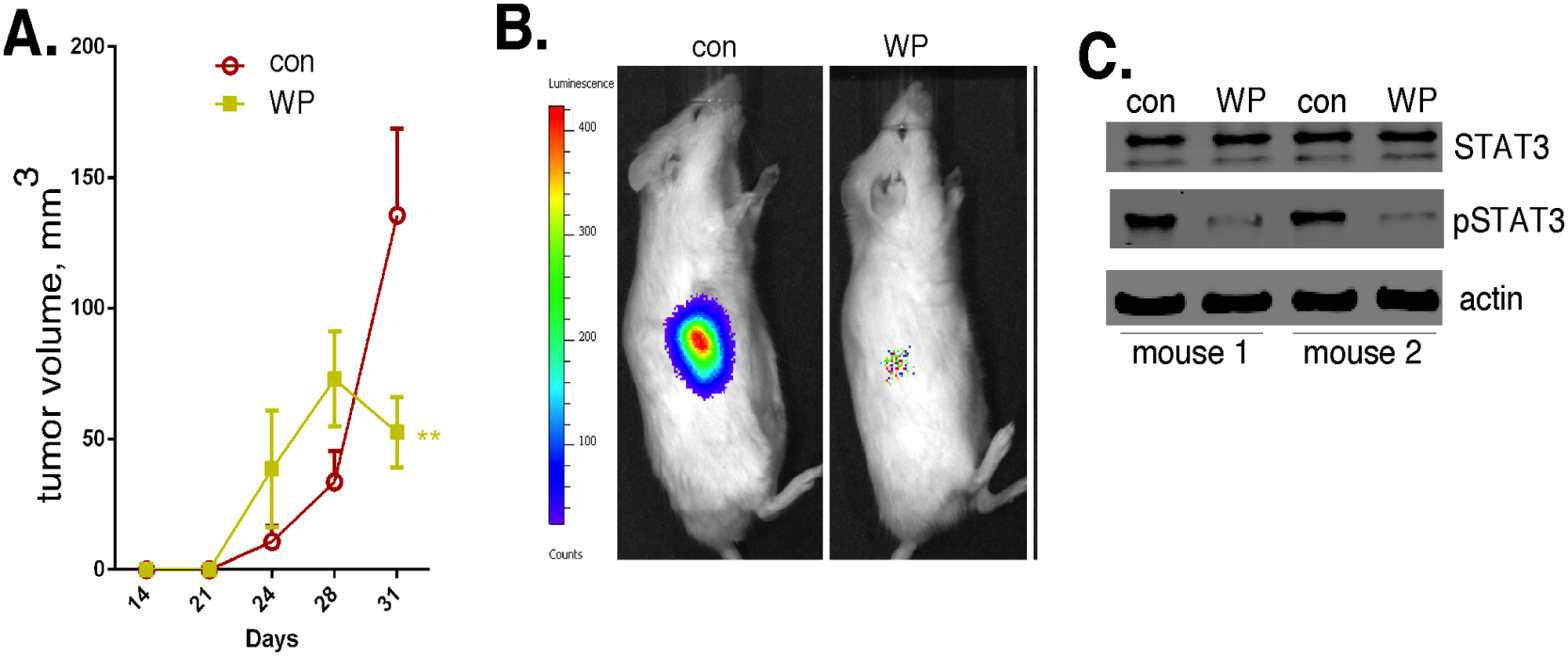
The antiglioma effects of the STAT3 inhibitor WP1066. Mice were subcutaneously injected with 1x10^6^ luciferase-tagged Ad-GSCs, and upon initial detection of tumor, mice were treated with WP1066 (40 mg/kg) every other day. A. Tumor volume was determined with calipers (n = 5). B. Representative bioluminescence images at Day 31. C. Lysates of tumor tissue at one week following WP1066 treatment were immunoblotted for STAT3 and phospho-STAT3 as indicated. Error bars, S.D. * p < 0.05, ** p < 0.01, *** p < 0.001.

We then examined the anticancer activity of the STAT3 inhibitor (WP1066) *in vivo*. NSG mice were flank injected with Ad-GSCs (1 x10^6^ cells) that express luciferase for noninvasive bioluminescence live animal imaging, and imaged twice a week. Once palpable tumors were detected (at ~ two weeks after tumor cell injection), WP1066 (40 mg/kg) was delivered intraperitoneally every other day for two weeks. As shown in Fig 9A, the tumorigenicity of Ad-GSCs was markedly suppressed by treatment with WP1066. The apparent increase in tumor size upon treatment with WP1066 at 24 and 28 days is apparently due to tumor necrosis, because live animal imaging showed a marked decrease in bioluminescence during this time after treatment (Fig 9B). In addition, immunoblotting of tumor tissue showed that high STAT3 activity in Ad-GSC xenografts was evident by virtue of Y705-STAT3 phosphorylation, and that WP1066 treatment of mice nearly abolished STAT3 activity (Fig 9C). These results demonstrate the anticancer activity of this STAT3 inhibitor against the Ad-GSC subpopulation *in vivo*.

## Discussion

The original name of glioblastoma multiforme is derived from the histopathologic description of the varied morphologic features of this tumor and the presence of heterogeneous cell populations within a single tumor, in which lesions with a high degree of cellular and nuclear polymorphism and numerous giant cells coexist with areas of high cellular uniformity [41]. Genomic profiling of glioblastoma samples in the TCGA database has previously identified four molecular subtypes (Classical, Neural, Proneural and Mesenchymal) based on robust gene expression, which may result from different cancer stem cell populations driving tumorigenesis [4]. We employed microarray analysis to molecularly characterize glioblastoma bulk tumor cells, Ad-GSCs and Sp-GSCs, as well as tumor xenografts of each cell population, which revealed that *in vitro* both Ad-GSCs and Sp-GSCs had similar molecular signatures that were clearly distinct from that of bulk tumor cells. Moreover, these GSC populations *in vitro* expressed high levels of “classical” stem cells markers (Nestin, Sox2 and CD133) and low levels of differentiation markers (βIII-tubulin and glial fibrillary acid protein) when compared to bulk tumor cells. Both GSC populations have high tumor initiating activity in immunocompromised mice by subcutanenous or intracranial injection, they form tumors comprised of the multiple cell types found in GBM, and are histologically indistinguishable. Thus, both Ad-GSCs and Sp-GSCs are “true” cancer stem cells, because they are able to self-renew and generate differentiated progeny that comprise the bulk tumor.

Xenografts of bulk tumor cells and Sp-GSCs by subcutaneous or intracranial injection were identified to be of the Classical molecular subtype, while xenografts of Ad-GSCs were of the Mesenchymal subtype. Nonetheless, these tumor xenografts were found to be all high-grade gliomas with no differences observed by H&E staining or marker expression (GFAP, S100, OLIG2, MAP2 and SYN). These findings reveal at the microscopic level the similarity between the molecularly distinct xenografts derived from bulk tumor cells, Sp-GSCs and Ad-GSCs. We performed intracranial injections of bulk tumor cells and GSCs to determine if the molecular heterogeneity found in our high-grade glioma xenografts is maintained in the conventional GBM microenvironment. The high tumor-initiating capacity of both Ad-GSCs and Sp-GSCs by intracranial injection was comparable to our previous studies performed by subcutaneous injection [25], and both GSC subpopulations formed tumors more rapidly than bulk tumor cells.

An important finding is that Ad-GSCs and Sp-GSCs tumor xenografts displayed distinct differences in their gene expression patterns. Both cancer stem cell populations (Ad-GSCs and Sp-GSCs) expressed Mesenchymal molecular signatures when grown *in vitro*. In contrast Ad-GSC tumor xenografts maintained a Mesenchymal glioblastoma gene signature, while tumors from Sp-GSCs expressed a Classical molecular signature like that of bulk tumor cells. These results were confirmed by qPCR of selective marker genes and GBM predictor genes [4]. For example, the GBM6 xenograft is derived from a patient with overexpression of the EGFRVIII mutant, and our finding of high EGFR expression in bulk tumor cells, and in tumors derived from them is consistent with EGFR overexpression. EGFR amplification is observed in 97% of Classical glioblastomas in the TCGA database but infrequently in other subtypes. However, it is extremely interesting that the Ad-GSC subpopulation and Ad-GSC tumor xenografts express relatively low EGFR levels providing additional evidence that Ad-GSCs are a distinct GSC population. The extensive heterogeneity at the cellular and molecular levels of glioblastoma that is replicated in GSC tumor xenografts has great significance, because this heterogeneity poses significant obstacles to the design of effective therapies [42]. Therefore, identification of a GSC subpopulation that exhibits a Mesenchymal gene signature *in vitro* and promotes the initiation and progression of the Mesenchymal glioblastoma subtype *in vivo* provides a valuable model of the human disease for future studies. Tumors with a Mesenchymal gene signature tend to be more aggressive, highly resistant to therapy, lead to a higher rate of relapse and have worse overall outcomes than other molecular subtypes of GBM [8]. In addition, while the expression of Mesenchymal marker genes was significantly elevated in intracranial Ad-GSCs tumor xenografts, Classical marker genes were highly expressed in intracranial xenografts of bulk tumor cells and Sp-GSCs. These orthotopic studies provide additional evidence that Ad-GSCs are a different tumor-initiating subpopulation than that of traditional Sp-GSCs. While both GSC subpopulations exhibit a Mesenchymal gene signature *in vitro*, Ad-GSCs maintain a Mesenchymal glioblastoma subtype when injected *in vivo* (intracranially and subcutaneously). In contrast, Sp-GSCs repopulate the tumor with a Classical gene signature similar to that of bulk tumor cells. Recent single cell analysis of GBM xenografts show that multiple molecular subtypes exist within a tumor and that this can even vary across individual cells [43]. Taken together, our results suggest that GSCs are not monoclonal but rather are a mosaic of different GSC populations, which are capable of initiating tumors with Mesenchymal and Classical gene signatures.

To identify novel potential therapeutic targets in the Ad-GSC Mesenchymal tumors, we performed a microarray analysis using a selective cancer-related gene panel and found a subset of genes whose expression was markedly different between xenografts of bulk tumor cells and Ad-GSCs. Most interestingly, STAT3 expression was elevated in the more aggressive Ad-GSC-derived tumors. We previously found Ad-GSCs and Sp-GSCs grown *in vitro* exhibit constitutively high STAT3 activity, which was not due to increased STAT3 gene expression or protein levels [25]. Since STAT3 is an important regulator of genes involved in various steps of tumor progression, we employed IPA analysis to identify pathways that STAT3 could potentially regulate within Ad-GSC xenografts to promote gliomagenesis, and found significant upregulation of several genes involved in the angiogenic pathway, notably ANGPTL4. While EGFR reportedly induces ANGPTL4 expression and promotes tumor angiogenesis in malignant gliomas [44], ANGPTL4 activation apparently is independent of EGFR upregulation in Mesenchymal glioblastoma xenografts. STAT3 has been shown to activate ANGPTL4 directly or indirectly through transcriptional regulation of VEGF and HIF-1 in various human cancers [45, 46], so we next examined the relationship of STAT3 and ANGPTL4 expression in GBM to tumor grade and patient survival. High STAT3 and ANGPTL4 levels were observed in high-grade (GBM) samples in the TCGA database when compared to normal brain and low-grade glioma. Consistent with our findings, STAT3 is among the most frequently activated oncogenic proteins in multiple solid tumor types, and is a predictor of poor prognosis in many malignancies including gliomas [47]. ANGPTL4 has been identified in hypoxia gene sets that predict poor outcome in multiple tumor types. In a study of several epithelial tumor types, ANGPTL4 levels increased as tumors progressed from local to metastatic disease [48, 49]. In our studies, we found a strong correlation between poor patient survival with enhanced expression of STAT3 and ANGPTL4 in GBM patients. Moreover, a direct correlation in expression levels of STAT3 and ANGPTL4 was found within human GBM patient samples. We also found that STAT3 binds to the ANGPTL4 promoter in Ad-GSCs and regulates ANGPTL4 expression. These results indicate that the co-expression of STAT3 and ANGPTL4 may have diagnostic and prognostic utility in GBM.

**Fig 10:**
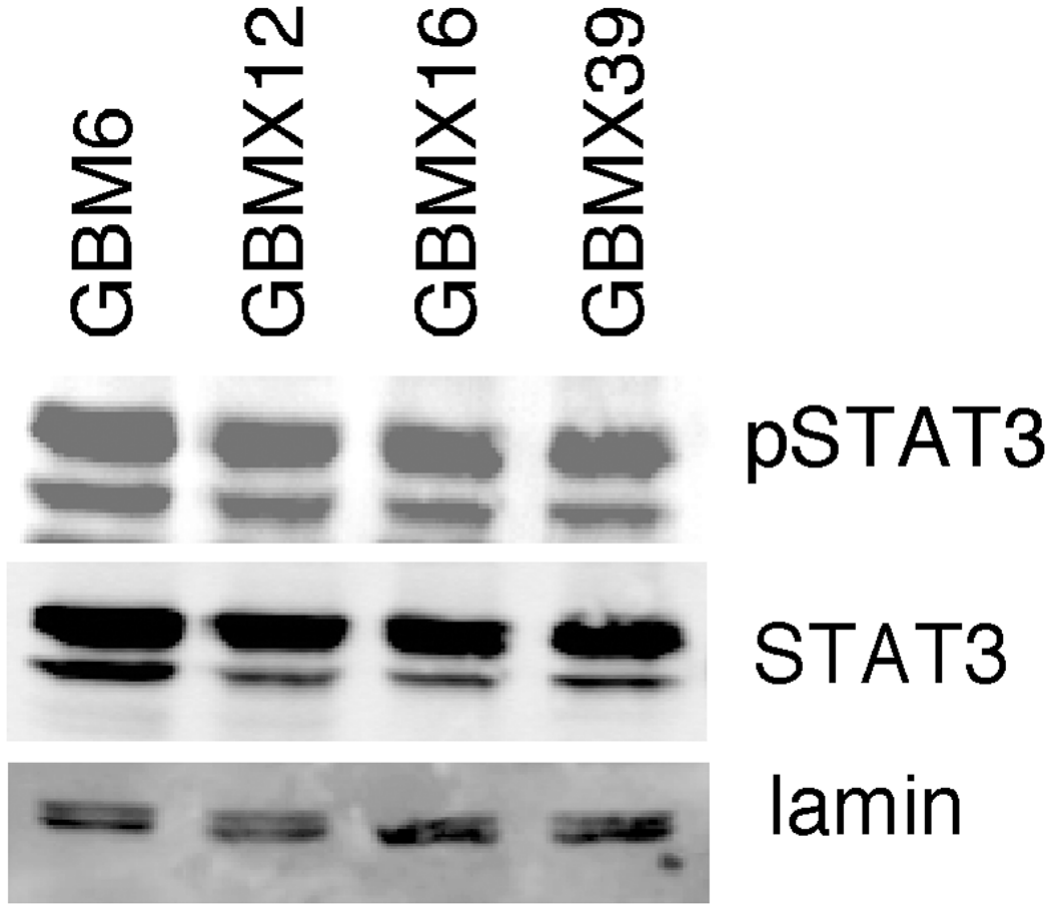
Constitutive activation of STAT3 in Ad-GSCs isolated from various GBM PDXs. The expression of STAT3 and pSTAT3 in nuclear extracts prepared from Ad-GSCs isolated from GBM6, GBMX12, GBMX16 and GBMX39 PDXs was determined by immunoblotting. Lamin expression served as a loading control.

We then examined the anticancer effects of the STAT3 inhibitor WP1066 on GSCs *in vitro* and *in vivo*. WP1066 is a JAK-2 kinase inhibitor that blocks STAT3 tyrosine phosphorylation, and has proapoptotic and antiproliferative activity in a variety of cancers, including glioma [21, 50]. We found that WP1066 decreased the expression of pro-angiogenic genes, including ANGPTL4, and ablated STAT3 binding to the ANGPTL4 promoter. WP1066 also inhibited the expression of stem cell marker genes. Most importantly, WP1066 decreased the tumorigenicity of Ad-GSCs and led to marked tumor regression. WP1066 has been shown to significantly inhibit growth of malignant glioma xenografts by blocking STAT3 activation and the subsequent induction of proliferation-related genes [21]. In preliminary studies we have found that high constitutive activation of STAT3 is observed in Ad-GSCs isolated from three additional GBM PDXs (Fig 10). Taken together, we have identified a significant relationship between STAT3 and ANGPTL4 in GBM stem cells and their therapeutic value as biomarkers in targeting the GSC subpopulation of Mesenchymal GBM.

## References

[1] Surawicz TS, Davis F, Freels S, Laws ER Jr, Menck HR. Brain tumor survival: results from the National Cancer Data Base. Journal of neuro-oncology. 1998;40(2):151–60. . 9892097 10.1023/a:1006091608586

[2] Stupp R, Mason WP, van den Bent MJ, Weller M, Fisher B, Taphoorn MJ, et al Radiotherapy plus concomitant and adjuvant temozolomide for glioblastoma. N Engl J Med. 2005;352(10):987–96. . 15758009 10.1056/NEJMoa043330

[3] Wong ET, Hess KR, Gleason MJ, Jaeckle KA, Kyritsis AP, Prados MD, et al Outcomes and prognostic factors in recurrent glioma patients enrolled onto phase II clinical trials. J Clin Oncol. 1999;17(8):2572–8. . 10561324 10.1200/JCO.1999.17.8.2572

[4] Verhaak RG, Hoadley KA, Purdom E, Wang V, Qi Y, Wilkerson MD, et al Integrated genomic analysis identifies clinically relevant subtypes of glioblastoma characterized by abnormalities in PDGFRA, IDH1, EGFR, and NF1. Cancer Cell. 2010;17(1):98–110. 10.1016/j.ccr.2009.12.020 20129251 PMC2818769

[5] Furnari FB, Fenton T, Bachoo RM, Mukasa A, Stommel JM, Stegh A, et al Malignant astrocytic glioma: genetics, biology, and paths to treatment. Genes Dev. 2007;21(21):2683–710. 10.1101/gad.1596707 . 17974913

[6] Watanabe K, Tachibana O, Sata K, Yonekawa Y, Kleihues P, Ohgaki H. Overexpression of the EGF receptor and p53 mutations are mutually exclusive in the evolution of primary and secondary glioblastomas. Brain pathology. 1996;6(3):217–23; discussion 23–4. . 8864278 10.1111/j.1750-3639.1996.tb00848.x

[7] Yan H, Parsons DW, Jin G, McLendon R, Rasheed BA, Yuan W, et al IDH1 and IDH2 mutations in gliomas. The New England journal of medicine. 2009;360(8):765–73. 10.1056/NEJMoa0808710 19228619 PMC2820383

[8] Phillips HS, Kharbanda S, Chen R, Forrest WF, Soriano RH, Wu TD, et al Molecular subclasses of high-grade glioma predict prognosis, delineate a pattern of disease progression, and resemble stages in neurogenesis. Cancer Cell. 2006;9(3):157–73. 10.1016/j.ccr.2006.02.019 . 16530701

[9] Zhu Y, Ghosh P, Charnay P, Burns DK, Parada LF. Neurofibromas in NF1: Schwann cell origin and role of tumor environment. Science. 2002;296(5569):920–2. 10.1126/science.1068452 11988578 PMC3024710

[10] Clarke MF, Dick JE, Dirks PB, Eaves CJ, Jamieson CH, Jones DL, et al Cancer stem cells—perspectives on current status and future directions: AACR Workshop on cancer stem cells. Cancer Res. 2006;66(19):9339–44. Epub 2006/09/23. 0008-5472.CAN-06-3126 [pii] 10.1158/0008-5472.CAN-06-3126 . 16990346

[11] Mani SA, Guo W, Liao MJ, Eaton EN, Ayyanan A, Zhou AY, et al The epithelial-mesenchymal transition generates cells with properties of stem cells. Cell. 2008;133(4):704–15. 10.1016/j.cell.2008.03.027 18485877 PMC2728032

[12] Marotta LL, Polyak K. Cancer stem cells: a model in the making. Current opinion in genetics & development. 2009;19(1):44–50. 10.1016/j.gde.2008.12.003 . 19167210

[13] Huntly BJ, Gilliland DG. Leukaemia stem cells and the evolution of cancer-stem-cell research. Nat Rev Cancer. 2005;5(4):311–21. . 15803157 10.1038/nrc1592

[14] Dontu G, Abdallah WM, Foley JM, Jackson KW, Clarke MF, Kawamura MJ, et al In vitro propagation and transcriptional profiling of human mammary stem/progenitor cells. Genes Dev. 2003;17(10):1253–70. . 12756227 10.1101/gad.1061803 PMC196056

[15] Lee A, Kessler JD, Read TA, Kaiser C, Corbeil D, Huttner WB, et al Isolation of neural stem cells from the postnatal cerebellum. Nat Neurosci. 2005;8(6):723–9. . 15908947 10.1038/nn1473 PMC2377345

[16] Pollard SM, Yoshikawa K, Clarke ID, Danovi D, Stricker S, Russell R, et al Glioma stem cell lines expanded in adherent culture have tumor-specific phenotypes and are suitable for chemical and genetic screens. Cell Stem Cell. 2009;4(6):568–80. Epub 2009/06/06. 10.1016/j.stem.2009.03.014 . 19497285

[17] Okochi-Takada E, Hattori N, Tsukamoto T, Miyamoto K, Ando T, Ito S, et al ANGPTL4 is a secreted tumor suppressor that inhibits angiogenesis. Oncogene. 2014;33(17):2273–8. 10.1038/onc.2013.174 . 23686315

[18] Tan MJ, Teo Z, Sng MK, Zhu P, Tan NS. Emerging roles of angiopoietin-like 4 in human cancer. Mol Cancer Res. 2012;10(6):677–88. 10.1158/1541-7786.MCR-11-0519 . 22661548

[19] Venkatesh HS, Chaumeil MM, Ward CS, Haas-Kogan DA, James CD, Ronen SM. Reduced phosphocholine and hyperpolarized lactate provide magnetic resonance biomarkers of PI3K/Akt/mTOR inhibition in glioblastoma. Neuro Oncol. 2012;14(3):315–25. 10.1093/neuonc/nor209 22156546 PMC3280799

[20] Yang CH, Yue J, Pfeffer SR, Handorf CR, Pfeffer LM. MicroRNA miR-21 Regulates the Metastatic Behavior of B16 Melanoma Cells. J Biol Chem. 2011;286(45):39172–8. Epub 2011/09/24. 10.1074/jbc.M111.285098 . 21940630 PMC3234742

[21] Iwamaru A, Szymanski S, Iwado E, Aoki H, Yokoyama T, Fokt I, et al A novel inhibitor of the STAT3 pathway induces apoptosis in malignant glioma cells both in vitro and in vivo. Oncogene. 2007;26(17):2435–44. 10.1038/sj.onc.1210031 . 17043651

[22] Horiguchi A, Asano T, Kuroda K, Sato A, Asakuma J, Ito K, et al STAT3 inhibitor WP1066 as a novel therapeutic agent for renal cell carcinoma. Br J Cancer. 2010;102(11):1592–9. 10.1038/sj.bjc.6605691 20461084 PMC2883159

[23] Kong LY, Abou-Ghazal MK, Wei J, Chakraborty A, Sun W, Qiao W, et al A novel inhibitor of signal transducers and activators of transcription 3 activation is efficacious against established central nervous system melanoma and inhibits regulatory T cells. Clin Cancer Res. 2008;14(18):5759–68. 10.1158/1078-0432.CCR-08-0377 18794085 PMC2583362

[24] Liang Y, Diehn M, Watson N, Bollen AW, Aldape KD, Nicholas MK, et al Gene expression profiling reveals molecularly and clinically distinct subtypes of glioblastoma multiforme. Proc Natl Acad Sci U S A. 2005;102(16):5814–9. 10.1073/pnas.0402870102 15827123 PMC556127

[25] Garner JM, Fan M, Yang CH, Du Z, Sims M, Davidoff AM, et al Constitutive Activation of Signal Transducer and Activator of Transcription 3 (STAT3) and Nuclear Factor kappaB Signaling in Glioblastoma Cancer Stem Cells Regulates the Notch Pathway. J Biol Chem. 2013;288(36):26167–76. 10.1074/jbc.M113.477950 23902772 PMC3764819

[26] Lee J, Kotliarova S, Kotliarov Y, Li A, Su Q, Donin NM, et al Tumor stem cells derived from glioblastomas cultured in bFGF and EGF more closely mirror the phenotype and genotype of primary tumors than do serum-cultured cell lines. Cancer Cell. 2006;9(5):391–403. . 16697959 10.1016/j.ccr.2006.03.030

[27] Thiery JP. Epithelial-mesenchymal transitions in tumour progression. Nat Rev Cancer. 2002;2(6):442–54. 10.1038/nrc822 . 12189386

[28] Bhat KP, Balasubramaniyan V, Vaillant B, Ezhilarasan R, Hummelink K, Hollingsworth F, et al Mesenchymal differentiation mediated by NF-kappaB promotes radiation resistance in glioblastoma. Cancer Cell. 2013;24(3):331–46. 10.1016/j.ccr.2013.08.001 23993863 PMC3817560

[29] Eng LF, Ghirnikar RS, Lee YL. Glial fibrillary acidic protein: GFAP-thirty-one years (1969–2000). Neurochemical research. 2000;25(9–10):1439–51. . 11059815 10.1023/a:1007677003387

[30] Kahn HJ, Marks A, Thom H, Baumal R. Role of antibody to S100 protein in diagnostic pathology. American journal of clinical pathology. 1983;79(3):341–7. . 6299096 10.1093/ajcp/79.3.341

[31] Hirokawa N, Hisanaga S, Shiomura Y. MAP2 is a component of crossbridges between microtubules and neurofilaments in the neuronal cytoskeleton: quick-freeze, deep-etch immunoelectron microscopy and reconstitution studies. The Journal of neuroscience: the official journal of the Society for Neuroscience. 1988;8(8):2769–79. . 3045269 10.1523/JNEUROSCI.08-08-02769.1988 PMC6569399

[32] Marie Y, Sanson M, Mokhtari K, Leuraud P, Kujas M, Delattre JY, et al OLIG2 as a specific marker of oligodendroglial tumour cells. Lancet. 2001;358(9278):298–300. 10.1016/S0140-6736(01)05499-X . 11498220

[33] Xie Q, Thompson R, Hardy K, DeCamp L, Berghuis B, Sigler R, et al A highly invasive human glioblastoma pre-clinical model for testing therapeutics. Journal of translational medicine. 2008;6:77 10.1186/1479-5876-6-77 19055779 PMC2645376

[34] Abdelzaher E. CNS Tumors Pathology—Glioblastoma Multiforme PathologyOutlines.com2013 [updated March 2012; cited 2014 March 15]. Available: http://www.pathologyoutlines.com/topic/cnstumorglioblastoma.html.

[35] Carro MS, Lim WK, Alvarez MJ, Bollo RJ, Zhao X, Snyder EY, et al The transcriptional network for mesenchymal transformation of brain tumours. Nature. 2010;463(7279):318–25. 10.1038/nature08712 . 20032975 PMC4011561

[36] Lewis-Tuffin LJ, Rodriguez F, Giannini C, Scheithauer B, Necela BM, Sarkaria JN, et al Misregulated E-cadherin expression associated with an aggressive brain tumor phenotype. PLoS One. 2010;5(10):e13665 10.1371/journal.pone.0013665 21060868 PMC2965143

[37] Liu WM, Huang P, Kar N, Burgett M, Muller-Greven G, Nowacki AS, et al Lyn facilitates glioblastoma cell survival under conditions of nutrient deprivation by promoting autophagy. PLoS One. 2013;8(8):e70804 10.1371/journal.pone.0070804 23936469 PMC3732228

[38] Okamura T, Kurisu K, Yamamoto W, Takano H, Nishiyama M. NADPH/quinone oxidoreductase is a priority target of glioblastoma chemotherapy. Int J Oncol. 2000;16(2):295–303. . 10639573 10.3892/ijo.16.2.295

[39] Brat DJ, Bellail AC, Van Meir EG. The role of interleukin-8 and its receptors in gliomagenesis and tumoral angiogenesis. Neuro-oncology. 2005;7(2):122–33. 10.1215/S1152851704001061 15831231 PMC1871893

[40] Strieter RM, Burdick MD, Mestas J, Gomperts B, Keane MP, Belperio JA. Cancer CXC chemokine networks and tumour angiogenesis. Eur J Cancer. 2006;42(6):768–78. 10.1016/j.ejca.2006.01.006 . 16510280

[41] Kleihues P, Sobin LH. World Health Organization classification of tumors. Cancer. 2000;88(12):2887 . 10870076 10.1002/1097-0142(20000615)88:12<2887::aid-cncr32>3.0.co;2-f

[42] Bonavia R, Inda MM, Cavenee WK, Furnari FB. Heterogeneity maintenance in glioblastoma: a social network. Cancer Res. 2011;71(12):4055–60. 10.1158/0008-5472.CAN-11-0153 21628493 PMC3117065

[43] Patel AP, Tirosh I, Trombetta JJ, Shalek AK, Gillespie SM, Wakimoto H, et al Single-cell RNA-seq highlights intratumoral heterogeneity in primary glioblastoma. Science. 2014;344(6190):1396–401. 10.1126/science.1254257 24925914 PMC4123637

[44] Katanasaka Y, Kodera Y, Kitamura Y, Morimoto T, Tamura T, Koizumi F. Epidermal growth factor receptor variant type III markedly accelerates angiogenesis and tumor growth via inducing c-myc mediated angiopoietin-like 4 expression in malignant glioma. Molecular cancer. 2013;12:31 10.1186/1476-4598-12-31 23617883 PMC3641008

[45] Niu G, Wright KL, Huang M, Song L, Haura E, Turkson J, et al Constitutive Stat3 activity up-regulates VEGF expression and tumor angiogenesis. Oncogene. 2002;21(13):2000–8. Epub 2002/04/18. 10.1038/sj.onc.1205260 . 11960372

[46] Semenza GL. Targeting HIF-1 for cancer therapy. Nat Rev Cancer. 2003;3(10):721–32. Epub 2003/09/18. 10.1038/nrc1187nrc1187 [pii]. . 13130303

[47] Abou-Ghazal M, Yang DS, Qiao W, Reina-Ortiz C, Wei J, Kong LY, et al The incidence, correlation with tumor-infiltrating inflammation, and prognosis of phosphorylated STAT3 expression in human gliomas. Clin Cancer Res. 2008;14(24):8228–35. Epub 2008/12/18. 10.1158/1078-0432.CCR-08-132914/24/8228 [pii]. 19088040 PMC2605668

[48] Verine J, Lehmann-Che J, Soliman H, Feugeas JP, Vidal JS, Mongiat-Artus P, et al Determination of angptl4 mRNA as a diagnostic marker of primary and metastatic clear cell renal-cell carcinoma. PLoS One. 2010;5(4):e10421 Epub 2010/05/11. 10.1371/journal.pone.0010421 20454689 PMC2861680

[49] Zhu P, Goh YY, Chin HF, Kersten S, Tan NS. Angiopoietin-like 4: a decade of research. Biosci Rep. 2012;32(3):211–9. Epub 2012/03/31. BSR20110102 [pii] 10.1042/BSR20110102 . 22458843

[50] Hussain SF, Kong LY, Jordan J, Conrad C, Madden T, Fokt I, et al A novel small molecule inhibitor of signal transducers and activators of transcription 3 reverses immune tolerance in malignant glioma patients. Cancer Res. 2007;67(20):9630–6. Epub 2007/10/19. 10.1158/0008-5472.CAN-07-1243 . 17942891

