# Correction: Molecular Heterogeneity in a Patient-Derived Glioblastoma Xenoline Is Regulated by Different Cancer Stem Cell Populations

**DOI:** 10.1371/journal.pone.0145052

**Published:** 2015-12-10

**Authors:** Jo Meagan Garner, David W. Ellison, David Finkelstein, Debolina Ganguly, Ziyun Du, Michelle Sims, Chuan He Yang, Rodrigo B. Interiano, Andrew M. Davidoff, Lawrence M. Pfeffer

Fig 5 is incorrect. There are duplicated panels. The authors have provided a corrected version here.

**Fig 5:**
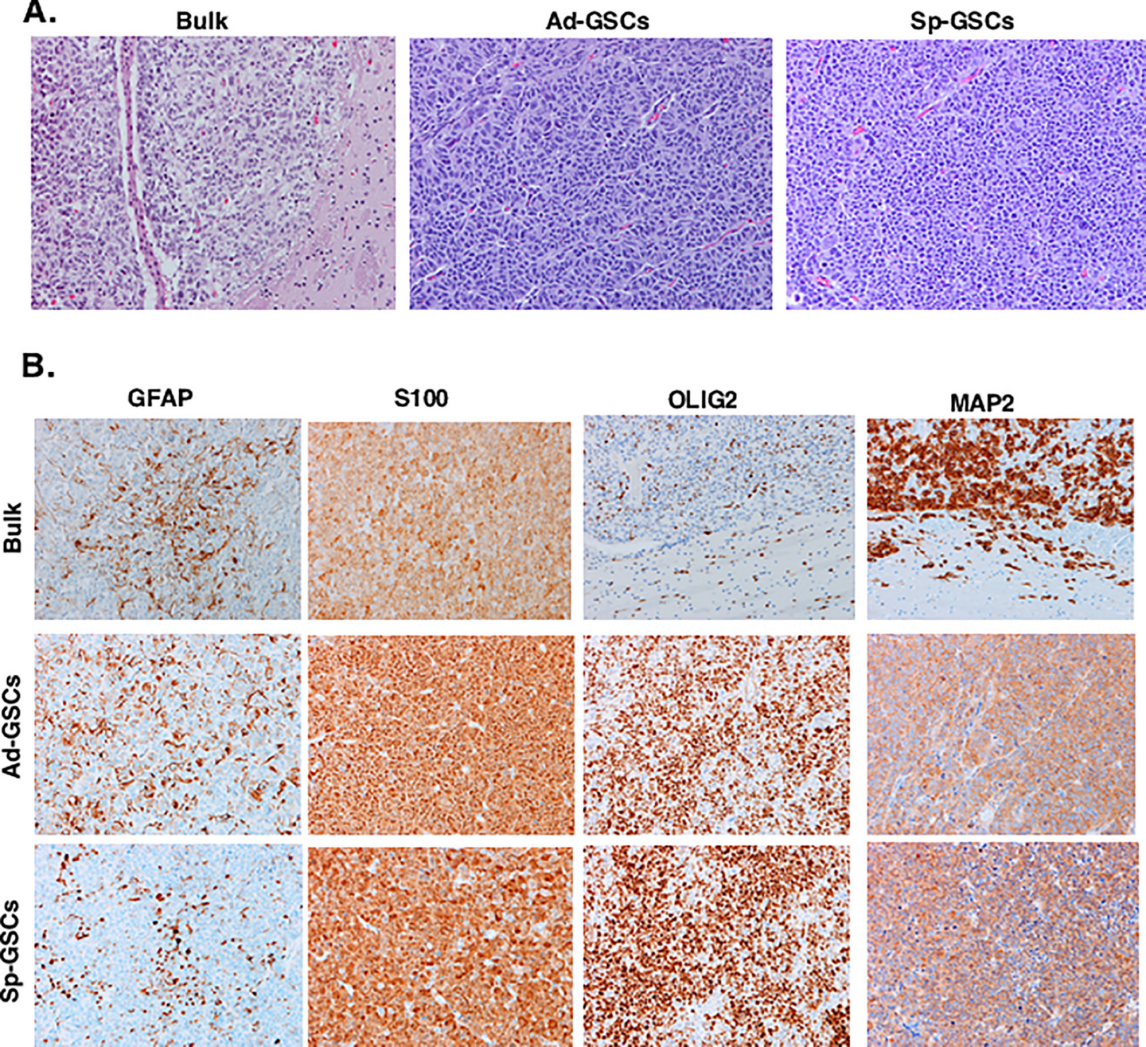
Pathologic analysis of orthotopic GBM6 tumor xenografts. GBM6 bulk tumor cells, Ad-GSCs and Sp-GSCs were orthotopically injected as described in Fig 4, and tumor-bearing brains were paraffin embedded for histology. A. H&E staining and B. Immunoreactivity for GFAP, S100, OLIG2 and MAP2. (All photomicrographs taken at 200x).
